# Exposure to Odors of Rivals Enhances Sexual Motivation in Male Giant Pandas

**DOI:** 10.1371/journal.pone.0069889

**Published:** 2013-08-05

**Authors:** Xiaoxing Bian, Dingzhen Liu, Hua Zeng, Guiquan Zhang, Rongping Wei, Rong Hou

**Affiliations:** 1 Key Laboratory of Biodiversity Science and Ecological Engineering of Ministry of Education, School of Life Sciences, Beijing Normal University, Beijing, China; 2 Key Laboratory for Reproduction and Conservation Genetics of Endangered Wildlife of Sichuan Province, China Conservation and Research Center for the Giant Panda, Wolong, Sichuan, China; 3 Key Laboratory for Conservation Biology of Endangered Wildlife of Sichuan Province, Chengdu Research Base of Giant Panda Breeding, Chengdu, Sichuan, China; Università degli Studi di Napoli Federico II, Italy

## Abstract

Males will alter their mating behavior to cope with the presence of their competitors. Even exposure to odors from potential competitors can greatly increase male ejaculate expenditure in a variety of animals including insects, fishes, birds and rodents. Major efforts have been made to examine males' plastic responses to sperm competition and its fitness benefits. However, the effects of competitor absence on male's sexual motivation and behaviors remain unclear, which has been proposed to be one of the causes for the poor sexual performance of some captive mammals. This study revealed that sexual motivation can be greatly enhanced in captive male giant pandas (*Ailuropoda melanoleuca*) by exposure to chemosensory cues from either one or three conspecifics males. It had been shown that potential rivals' odors increased males' chemosensory investigation behavior, as well as their observing, following and sniffing behaviors towards estrous females. Behaviors changed regardless of the number of rivals (one or three). Our results demonstrate the effects of potential competition on male giant pandas' sexual motivation and behavioral coping strategy. We anticipate that our research will provide a fresh insight into the mechanisms underlying poor sexual performance in male captive mammals, and valuable information for the practical management and *ex situ* conservation of endangered species.

## Introduction

Male-male competition for access to females plays an important role in reproductive success [1]. Such competition includes direct physical confrontations between individuals, as well as indirect confrontation via chemosensory and/or auditory cues. According to sexual selection theory, males can enhance their reproductive fitness in a rapidly changing environment by responding effectively to the presence and number of mating rivals [2]. Several empirical studies, mostly on insects and small vertebrates, have shown that males alter their mating behavior in the presence of rivals [2]. Exposure to chemosensory cues from conspecific males can greatly increase male reproductive investment in insects [3]–[5], fish [6], birds [7] and rodents [8], [9]. Unfortunately, little is known about the effects of the presence of conspecifics, or chemosensory cues from them, on behaviors of large mammals, partly because the experimental procedures involved in collecting the data are difficult.

The giant panda (*Ailuropoda melanoleuca*) is an endangered Chinese mammal which has become a flagship species for animal conservation world-wide. The successful captive breeding program has resulted in a current captive population of about 333 individuals in 58 institutions around the world [10]. However, the low percentage (around 26%) of adult males that mate naturally in captivity has been a concern [11], and natural mating only accounts for about 40% of successful breeding in captive giant pandas (Zhi Huang, unpublished data). Lack of sexual motivation in males seems to be the primary cause of failure [12], and has seriously impaired the captive breeding program and the quality and genetic diversity of the captive population. It has been suggested that, among other factors, the unnatural social structure and the lack of intra-sex competition may contribute to poor reproductive performance in captive mammals [13].

Giant pandas can discriminate other individuals via chemosensory cues from urine and anogenital gland secretions (AGS) [14]–[19]. During the breeding season in the wild, male pandas usually use olfactory and auditory signals to follow estrous females, and compete with each other for first access to females [20]. However, captive pandas are housed individually, and are not usually exposed to rivals during the mating season. In the present study, we exposed male giant pandas to chemosensory cues from conspecific males, and examined their subsequent behavior towards neighboring females. We hypothesized that chemosensory cues from potential rivals may increase male pandas' sexual motivation towards females, and enhance their territorial behavior.

## Materials and Methods

### Subjects and housing conditions

Subjects were housed in the Breeding Center at the China Conservation and Research Center for the Giant Panda (CCRCGP), Bifengxia Base, Sichuan, People's Republic of China, individually in pens consisting of an outdoor yard (6×10 m; Figure S1a) and an indoor bedroom (6×4 m). Male and female giant pandas were housed in alternate pens during the mating season, and were fed on bamboo, shoots, panda bread (containing nutritional supplements), apples and carrots.

Eleven genetically unrelated adult male giant pandas ages 6–17 years (studbook # 424, 455, 488, 502, 503, 542, 579, 586, 595, 623 and 661), chosen for breeding in 2012 by the Giant Panda Annual Breeding Committee, were used as subjects. Estrous females were moved to the breeding center and kept between two candidate males to promote familiarization. Males and adjacent estrous females were able to maintain visual, olfactory, acoustic and limited tactile communication through the fences (Figure S1d).

Odor-exposure trials were executed when neighboring females began to show typical estrous behaviors such as chirps, increased bleats and scent marking, but before tail-up displays were observed. This state usually lasts for 5–10 days and is followed by the female's estrus peak. The first trial on each subject was conducted approximately two days after he was acclimatized to the breeding center. All trials were carried out when pandas were active, from 0700–0830 h in March and April 2012. Our experiments were carried out within the regulations of CCRCGP and adhered to the Chinese Regulations and Standards for Captive Animals. All animal use and management protocols were approved by the Institutional Animal Care and Use Committee of Beijing Normal University.

### Odor source collection and exposure

The stimulus males, used as donors, were selected randomly. Fresh urine and fecal samples were collected in test tubes or sealable bags. Samples of fresh AGS were collected by rubbing pieces of bark from fir trees (*Abies faxoniana*, used to enrich the enclosure's environment; 6×8 cm) against the surface on which the stimulus male had deposited AGS. Odor sources were collected the day before the trial and stored in a refrigerator at 4°C. Multiple odor sources were used to mimic the “scent posts” of pandas in the wild [20].

Each subject was randomly assigned to one of three experimental groups that were exposed to the odor sources from a single male (SM) or from multiple males (MM), or were used as a control (Control). In each trial, five odor sources were placed in the subject's yard (Figure S1a): two fresh fecal samples were placed in the center of the yard, and four pieces of bark, two with AGS and two with urine, were placed alternately in the corners of the yard, either hung on the fence (0.5–1.0 m above the ground) or placed on the ground. For the SM group, odor sources in one trial were from the same donor, while for the MM group, odor sources were from three donors. The control males were presented with four pieces of untreated bark and fecal samples of their own. The experiment was conducted using a repeated measures design; each subject was assigned to one of the three treatment groups in a randomized order every other day.

### Behavioral quantification

After the odor sources had been put in place, each subject was released into the yard and its behavior was recorded continuously [21] by a digital camera (Sony DCR-SR68E, Sony, Shanghai, China). Detailed observation started when the subject found and sniffed any of the five scent sources (including untreated bark), and lasted for 30 minutes. The videos were played back and scored using Observer XT9 (Noldus, the Netherlands).

The durations of the following behaviors were quantified: chemosensory investigation (the subject placed its nose within 5 cm of a surface and showed nasal movement accompanied by inhalation); observing female (the subject watched a neighboring female and followed her movement with eye movements; Figure S1c); following female (the subject kept within two body-lengths of a female; Figure S1e); and sniffing female (the subject placed his nose within 5 cm of a female's body and showed nasal movement accompanied by inhalation; Figure S1f). The frequency of occurrence of territorial behavior was recorded, including urination (depositing a small amount of urine on the ground) and AGS scent marking (rubbing the anogenital region on objects). In addition, the frequency of occurrence of body rubbing (rubbing the scruff against a surface), bleating (making the sheep-like call which is the most common form of acoustic communication in giant pandas), and foot scraping (scraping the substrate with a backward motion of the hind paws) were recorded.

### Data Analysis

For chemosensory investigation and observing females, we used absolute durations for analyses. For sniffing and following females, durations were divided by the time spent by the neighboring females near the fence, to calculate the proportion (percentage) of available time spent on these behaviors. Data on chemosensory investigation, observing females, urination, AGS scent-marking, body rubbing, bleating and foot scraping were normally distributed and were analyzed using repeated measures analysis of variance (ANOVA) followed by post hoc paired-sample t-tests. The data on sniffing females were normally distributed after arcsine transformation and were analyzed in the same way. The data on following females were not normally distributed and were analyzed using Friedman one-way tests followed by post hoc Wilcoxon sign rank tests.

Observing, following, and sniffing females were subsequently combined as “female-oriented behavior” and used to quantify the males' sexual motivation. To reveal differences between treatment groups in temporal patterns of behavioral changes, we quantified chemosensory investigation (normally distributed) and female-oriented behavior (normally distributed after square root transformation) at 2-min intervals during the 30-min observation periods, and submitted the data to repeated measures ANOVA (treatment × minutes). Data on these two behaviors in each 2-min interval were analyzed by using repeated measures ANOVAs followed by paired-sample t-tests. All statistical analyses were carried out using SPSS 18.0 for Windows (SPSS Inc., Chicago, USA).Data are shown as mean +/− SE (standard error), alpha was set at 0.05, and all tests were two-tailed.

## Results

**Figure 1:**
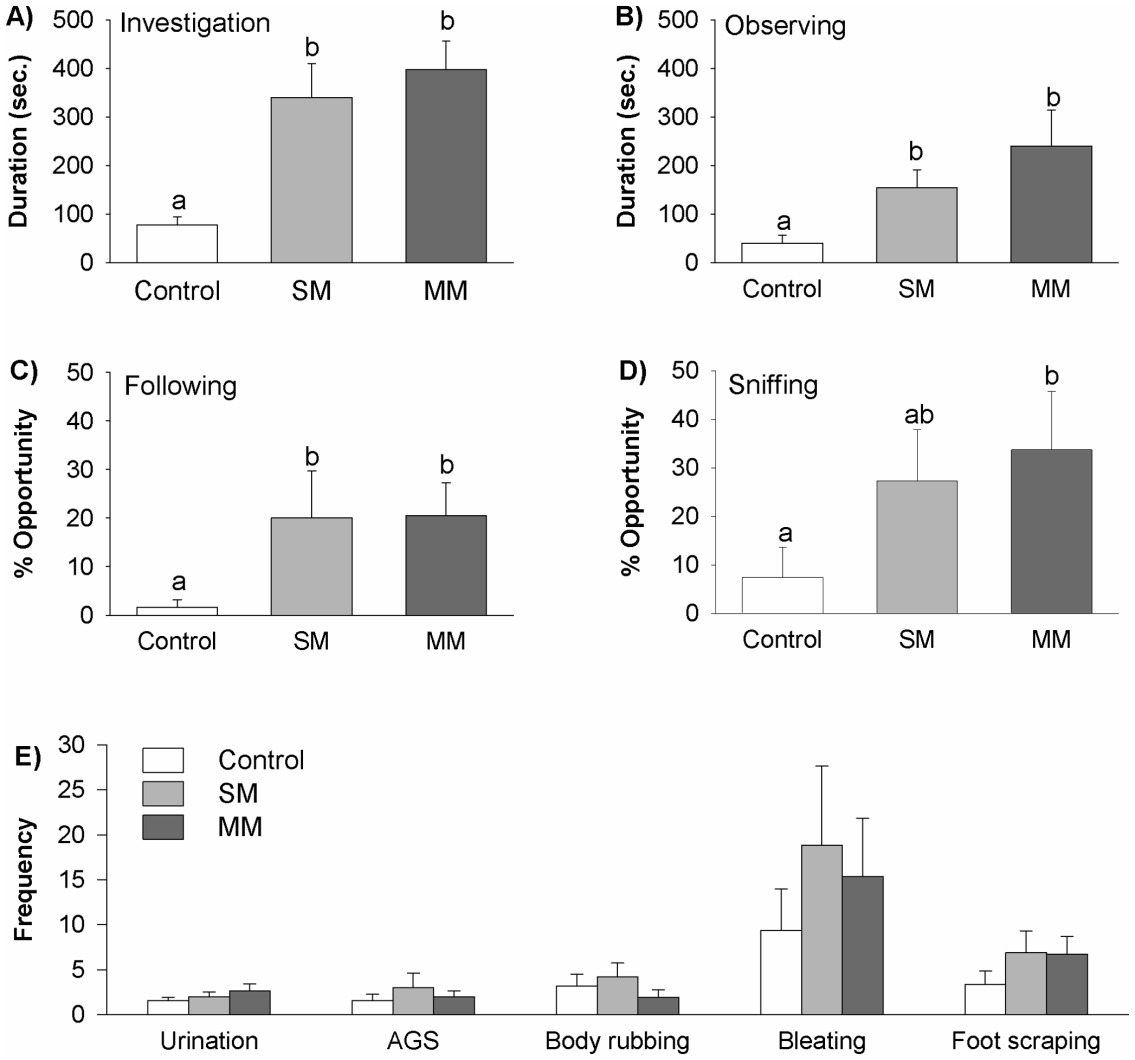
Behaviors of male pandas in different treatment groups. Males exposed to odor sources from either a single male (SM) or multiple males (MM) spent more time on A) chemosensory investigation as well as on B) observing and C) following neighboring females than Control males, and MM males D)sniffed females more than Control males. E) No differences between groups were found in the frequency of other behaviors including urination, AGS scent-marking, body rubbing, bleating, and foot scraping. Data are mean + SE, bars with different letters differ significantly from each other.

The presence of odor sources from stimulus male pandas led to significant increases in the time subjects in both SM and MM experimental groups spent in chemosensory investigation of their environment (*F*_1,10_ = 15.623, *P*<0.001; Figure 1a). Group differences were also found in the amount of time subjects spent on observing (*F*_1,10_  = 4.616, *P* = 0.022; Figure 1b, S1c), following ( *χ^2^* = 6.690, df  = 2, *P* = 0.035; Figure 1c, S1e), and sniffing (*F*_1,10_ = 4.443, *P* = 0.025; Figure 1d, S1f) the females. Post-hoc tests indicated that males in both SM and MM groups observed and followed the females significantly more than control males, and that MM males sniffed females more than control males. No significant differences were found between SM and MM males, and no differences between groups were found in the frequencies of urination (*F*_1,10_ = 1.320, *P* = 0.290), AGS scent-marking (*F*_1,10_ = 0.511, *P* = 0.608), body rubbing (*F*_1,10_ = 0.717, *P* = 0.500), bleating (*F*_1,10_ = 0.789, *P* = 0.468) or foot scraping (*F*_1,10_ = 2.183, *P* = 0.139; Figure 1e).

**Figure 2:**
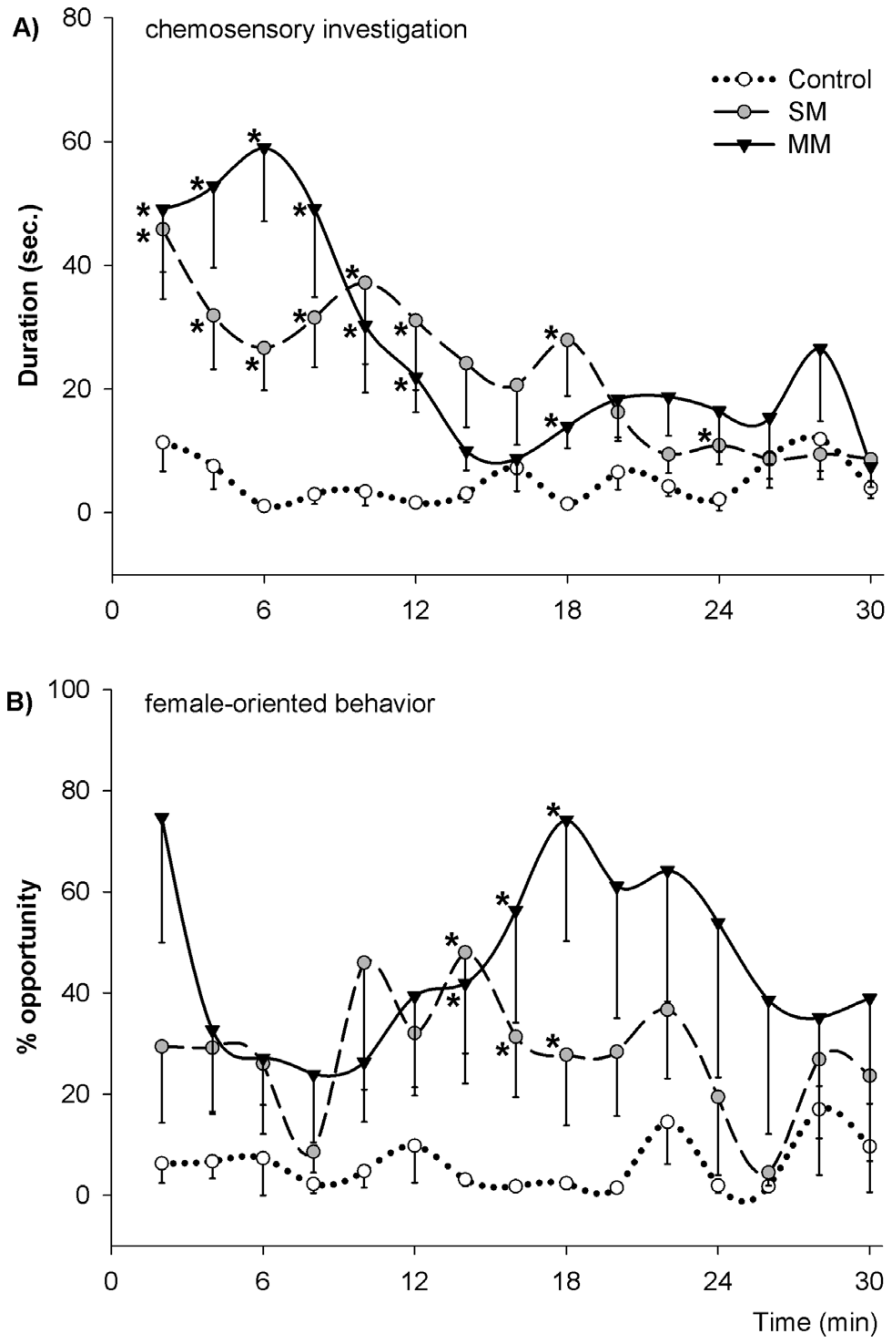
Temporal patterns of behavior in male pandas. A)Chemosensory investigation and female-oriented behavior (including observing, following, and sniffing the females; B) in male giant pandas that were exposed to odor sources from either a single male (SM) or multiple males (MM), or to their own fecal samples (Control) within 30 minutes of the observation period. Data are shown as mean – SE; * indicates a significant difference from the control.

Exposure to odor sources from conspecific males led to changes in temporal patterns of chemosensory investigation and female-oriented behavior (including observing, following, and sniffing the females). Males in both SM and MM groups spent more time on chemosensory investigation behavior than control males (*F*_2, 30_ = 10.198, *P*<0.001). With some fluctuations, this behavior gradually decreased to the level of control males 12 min after exposure to odor sources from conspecific males (Figure 2a). Furthermore, males in both SM and MM groups displayed increased levels of female-oriented behavior (*F*_2, 30_ = 7.514, *P* = 0.002), especially from 12 to 18 min after exposure (Figure 2b). Neither chemosensory investigation nor female-oriented behavior showed different levels between SM and MM groups. Control males did not display significant changes in the temporal patterns of either behavior. Typically, males in the SM and MM groups switched from chemosensory investigation to female-oriented behavior after around 12 minutes of exposure to odor sources from other males (Figure 2a, b).

## Discussion

Chemosensory cues play an important role in animals' social behavior [22] and are thought to be involved in sexual selection through their role in mate choice, competition for mates, or defense of resources needed to attract mates [23]. Male compete for access to females with one another, either via direct physical confrontations or indirectly via chemosensory and/or auditory cues. Male-male competition may lead to significant behavioral plasticity and thus increased reproductive fitness [2], [5]. In the present study, we found that odors of conspecific males led to increases in male giant pandas' chemosensory investigation behavior and in behaviors displayed towards neighboring estrous females. These effects were specific to sexual behavior: no group differences were found in territorial or other behaviors.

Exposure to conspecific male odors in combination with the presence of estrous females elicited a high level of chemosensory investigation, indicating increased arousal in male giant pandas. Subsequently, these males displayed enhanced female-oriented behaviors including observing, following, and sniffing the neighboring females, indicative of increased sexual motivation. These data are consistent with previous studies in a broad range of animals [3], [4], [6]–[8]. However, it is remarkable that male pandas alter their behavior before any courtship behavior has taken place, under the influence of perceived male-male competition. Giant pandas are capable of discriminating between different individuals via chemosensory cues [14]. Therefore, lack of behavioral differences between SM and MM males should not be interpreted as a lack of individual recognition. Instead, chemosensory cues from a single individual were sufficient to affect behavior, and cues from multiple individuals did not have synergistic effects on male pandas' behavior. This phenomenon was previously reported in fruit flies [24], and yet does not conform to the intensity model of sperm competition [25]. Our temporal analysis (Figure 2) demonstrated that at a switch point 12 minutes after exposure males may switch from investigating the environment to showing interest in females.

One strategy in male-male competition is to defend a territory and resources which may be attractive to potential mates [26]–[28]. In our study, however, exposure to conspecific male odor had no effect on typical territorial behaviors such as urination and AGS scent-marking, or on other behaviors, such as body rubbing, foot scraping, and bleating, that may be related to communication and aggressiveness [29], [30]. These data suggest that male pandas prefer mate guarding, as indicated by observing and following females, to territory guarding in male-male competition. In the wild, adult male pandas usually chase estrous females and fight with each other for mating rights, rather than defending territories [31]. Scent marking takes place both within and outside the breeding season [32]. Giant pandas are highly promiscuous [20], and the females' fertile period lasts for only 1–3 days each year [32]. Therefore, it might be a better strategy for male giant pandas to locate and guard estrous females than to guard territories, as opportunities to fertilize females are rare [33].

Our data indicate that chemosensory cues from potential competitors can cause arousal in male giant pandas, leading to an increase in sexual motivation and sexual behavior. As intra-male competition is critical to social structure in giant pandas in the wild [20], [33], our data suggest that exposing males to the odor of other males in addition to facilitating hetero-sex chemical communication [15] may be a useful technique to enhance captive breeding in this endangered species. Needless to say, whether increased sexual motivation actually facilitates mating and leads to improve reproductive success requires further study.

## Supporting Information

Figure S1 **Photos and drawings illustrating the behavior of captive giant pandas.** (a) A male panda in the outdoor yard of its enclosure. Four pieces of bark (I – IV) containing alternately urine or AGS were either placed on the ground or hung on the fence, at the corners of the yard. Droppings were placed in the center (V) of the yard; (b) a male panda sniffing bark containing chemosensory stimuli and (c) observing a female; (d) a male (foreground) and a female panda observing and sniffing each other through the fence; (e) a male panda following the neighboring female.
(TIFF)

## References

[1] Darwin C (2010) The descent of man: New York: Dover Publications Inc.

[2] Bretman A, Gage MJG, Chapman T (2011) Quick-change artists: male plastic behavioural responses to rivals. Trends Ecol Evol 26: 467–473. 21680050 10.1016/j.tree.2011.05.002

[3] Wedell N, Cook PA (1999) Butterflies tailor their ejaculate in response to sperm competition risk and intensity. Proc R Soc Lond B Biol Sci 266: 1033–1039.

[4] Gage MJG (1991) Risk of sperm competition directly affects ejaculate size in the mediterranean fruit fly. Anim Behav 42: 1036–1037.

[5] Bretman A, Westmancoat JD, Gage MJG, Chapman T (2011) Males use multiple, redundant cues to detect mating rivals. Curr Biol 21: 617–622. 21439827 10.1016/j.cub.2011.03.008

[6] Pilastro A, Scaggiante M, Rasotto MB (2002) Individual adjustment of sperm expenditure accords with sperm competition theory. Proc Natl Acad Sci U S A 99: 9913–9915. 12107282 10.1073/pnas.152133499 PMC126598

[7] Pizzari T, Cornwallis CK, Lovlie H, Jakobsson S, Birkhead TR (2003) Sophisticated sperm allocation in male fowl. Nature 426: 70–74. 14603319 10.1038/nature02004

[8] delBarco-Trillo J, Ferkin MH (2004) Male mammals respond to a risk of sperm competition conveyed by odours of conspecific males. Nature 431: 446–449. 15386011 10.1038/nature02845

[9] Vaughn AA, Ferkin MH (2011) The presence and number of male competitor's scent marks and female reproductive state affect the response of male meadow voles to female conspecifics' odours. Behaviour 148: 927–943.

[10] Xie Z (2011) The 2011 International Studbook For Giant Panda, *Ailuropoda melanoleuca*. Beijing: Chinese Association of Zoological Gardens. 261 p.

[11] Hu J (1990) Studies on reproductive ecology of the giant panda. In: Hu J, Wei F, Yuan C, Wu Y, editors. Research and progress in biology of the giant panda. Chengdu: Sichuan Publishing House of Science & Technology. 309–315.

[12] Zhang G, Swaisgood RR, Zhang H (2004) Evaluation of behavioral factors influencing reproductive success and failure in captive giant pandas. Zoo Biol 23: 15–31.

[13] Lindburg DG, Fitch-Snyder H (1994) Use of behavior to evaluate reproductive problems in captive mammals. Zoo Biol 13: 433–445.

[14] Swaisgood RR, Lindburg DG, Zhou X (1999) Giant pandas discriminate individual differences in conspecific scent. Anim Behav 57: 1045–1053. 10328790 10.1006/anbe.1998.1070

[15] Swaisgood RR, Lindburg DG, Zhou X, Owen MA (2000) The effects of sex, reproductive condition and context on discrimination of conspecific odours by giant pandas. Anim Behav 60: 227–237. 10973725 10.1006/anbe.2000.1464

[16] White AM, Swaisgood RR, Zhang H (2002) The highs and lows of chemical communication in giant pandas (*Ailuropoda melanoleuca*): effect of scent deposition height on signal discrimination. Behav Ecol Sociobiol 51: 519–529.

[17] White AM, Swaisgood RR, Zhang H (2003) Chemical communication in the giant panda (*Ailuropoda melanoleuca*): the role of age in the signaller and assessor. J Zool 259: 171–178.

[18] Yuan H, Liu DZ, Sun LX, Wei RP, Zhang GQ, et al (2004) Anogenital gland secretions code for sex and age in the giant panda, *Ailuropoda melanoleuca* . Can J Zool 82: 1596–1604.

[19] Zhang JX, Liu DZ, Sun LX, Wei RP, Zhang GQ, et al (2008) Potential chemosignals in the anogenital gland secretion of giant pandas, *Ailuropoda melanoleuca*, associated with sex and individual identity. J Chem Ecol 34: 398–407. 18293041 10.1007/s10886-008-9441-3

[20] Schaller GB, Hu J, Pan W, Zhu J (1985) The giant pandas of Wolong: University of Chicago Press Chicago, Illinois, USA.

[21] Martin P, Bateson P (1986) Measuring behaviour. An introductory guide. Cambridge University Press, Cambridge.

[22] Brown RE, Macdonald DW (1985) Social odours in mammals: Clarendon Press Oxford.

[23] Andersson M (1994) Sexual selection. Princeton, New Jersey: Princeton University Press.

[24] Bretman A, Fricke C, Hetherington P, Stone R, Chapman T (2010) Exposure to rivals and plastic responses to sperm competition in *Drosophila melanogaster* . Behav Ecol 21: 317–321.

[25] Parker GA, Ball MA, Stockley P, Gage MJG (1997) Sperm competition games: a prospective analysis of risk assessment. Proc R Soc Lond B Biol Sci 264: 1793–1802. 10.1098/rspb.1997.0249 PMC1688741 9447737

[26] Williams JM, Oehlert GW, Carlis JV, Pusey AE (2004) Why do male chimpanzees defend a group range? Anim Behav 68: 523–532.

[27] Waterman J (2007) Male mating strategies in rodents. In: Wolff JO, Sherman PW, editors. Rodent societies: an ecological and evolutionary perspective. 27–41.

[28] Hasegawa M, Arai E, Watanabe M, Nakamura M (2012) Female mate choice based on territory quality in barn swallows. J Ethol 30: 143–150.

[29] Ferkin MH (2007) Effects of previous interactions and sex on over-marking in meadow voles. Behaviour 144: 1297–1313.

[30] Kleiman DG, Karesh WB, Chu PR (1979) Behavioural changes associated with oestrus in the giant panda. International Zoo Yearbook 19: 217–224.

[31] Yong YG, Wei FW, Ye X, Zhang Z, Li Y (2004) Mating behaviors of wild giant pandas in Foping Natural Reserve. Acta Theriologica Sinica 24: 346–349.

[32] Kleiman DG (1983) Ethology and reproduction of captive giant pandas (*Ailuropoda melanoleuca*). Z Tierpsychol 62: 1–46.

[33] Nie YG, Swaisgood RR, Zhang ZJ, Liu XB, Wei FW (2012) Reproductive competition and fecal testosterone in wild male giant pandas (*Ailuropoda melanoleuca*). Behav Ecol Sociobiol 66: 721–730.

